# A mechanistic overview of approaches for the treatment of psychostimulant dependence

**DOI:** 10.3389/fphar.2022.854176

**Published:** 2022-09-08

**Authors:** Kathrine Louise Jensen, Søren Brøgger Jensen, Kenneth Lindegaard Madsen

**Affiliations:** Molecular Neuropharmacology and Genetics Laboratory, Department of Neuroscience, Faculty of Health and Medical Sciences, University of Copenhagen, Copenhagen, Denmark

**Keywords:** psychostimulant addiction, addiction treatment, cocaine dependence treatment, amphetamine dependence treatment, clinical trial, addiction disorders, psychostimulant dependence

## Abstract

Psychostimulant use disorder is a major health issue around the world with enormous individual, family-related and societal consequences, yet there are no effective pharmacological treatments available. In this review, a target-based overview of pharmacological treatments toward psychostimulant addiction will be presented. We will go through therapeutic approaches targeting different aspects of psychostimulant addiction with focus on three major areas; 1) drugs targeting signalling, and metabolism of the dopamine system, 2) drugs targeting either AMPA receptors or metabotropic glutamate receptors of the glutamate system and 3) drugs targeting the severe side-effects of quitting long-term psychostimulant use. For each of these major modes of intervention, findings from pre-clinical studies in rodents to clinical trials in humans will be listed, and future perspectives of the different treatment strategies as well as their potential side-effects will be discussed. Pharmaceuticals modulating the dopamine system, such as antipsychotics, DAT-inhibitors, and disulfiram, have shown some promising results. Cognitive enhancers have been found to increase aspects of behavioural control, and drugs targeting the glutamate system such as modulators of metabotropic glutamate receptors and AMPA receptors have provided interesting changes in relapse behaviour. Furthermore, CRF-antagonists directed toward alleviating the symptoms of the withdrawal stage have been examined with interesting resulting changes in behaviour. There are promising results investigating therapeutics for psychostimulant addiction, but further preclinical work and additional human studies with a more stratified patient selection are needed to prove sufficient evidence of efficacy and tolerability.

## Introduction

Addiction to psychostimulants is a world-wide health problem causing major societal problems, and still there is no efficient treatment available to cure addiction of these drugs. In 2010 it was estimated that 24.1 million people were dependent on psychostimulant drugs worldwide: 6.9 million people were cocaine-dependent, 17.2 million people were dependent on amphetamines (Degenhardt et al., 2014). Only 8 years later, in 2018, it was estimated that approximately 18.2 million people used cocaine and 34.2 million people used amphetamines (Farrell et al., 2019). Addiction is encompassed by the term substance use disorders and can be defined as a chronic and relapsing neuropsychiatric disorder characterized by drug seeking, high intake of drugs, and cycles of abstinence and relapse (Koob & Volkow, 2016). In the DSM-V, substance use disorder is defined as a chronically relapsing disorder with abuse symptoms such as hazardous use, legal problems, and craving, alongside dependence symptoms including withdrawal, tolerance, and repeated attempts to quit and control use.

Through animal models and brain imaging studies the neurobiology underlying addiction is being mapped, and although complex the progress made in this field of research has helped to increase the knowledge of the neurobiological adaptations driving addiction. Identifying specific changes in neurocircuitry and neuroplasticity have helped the understanding of addiction as a neuropsychiatric brain disease of learning and memory as opposed to the traditional model of self-determination and personal responsibility (Volkow & Koob, 2015; Volkow et al., 2016). Nevertheless, the social and psychological aspects of this disease should not be overlooked and a balanced perspective in addiction treatment is needed (Hall et al., 2015; Lewis, 2018).

Since psychostimulant addiction is a complex disorder, there are multiple aspects of the disease, which would make sense to target with pharmaceuticals. One approach would be to decrease consumption by minimizing the rewarding effects of the psychostimulants, thereby potentially altering the habit formation induced by the drugs. Another approach could be to interfere with the extreme abstinence-induced side-effects, to prevent the compulsive drug-seeking, thereby minimizing the motivational and emotional symptoms. Furthermore, reversing the disruption of executive functions of individuals with addiction disorder should increase an individual’s ability to stop/control drug use thereby preventing relapse behaviour. Relapse and craving might also be halted by interacting with the neurocircuits known to be involved in drug-, cue-, stress-, and context-induced reinstatement, thereby introducing an anti-craving state. During this review, we will touch upon drugs targeting all these aspects.

Due to the known aetiology of psychostimulant addiction, and decent construct and face validity of preclinical models, these are a great asset to reveal new pharmacotherapeutic strategies of this disorder specifically (Everitt et al., 2018). Therefore, our review includes both preclinical and clinical studies. There are obvious limitations of translating findings in rodents to the human condition, both due to species differences in general, but probably more importantly due to the complexity of the social aspects of addiction in humans as well as greater homogeneity of the human condition. However, instead of compromising the use of all preclinical setups, this highlights the need to distinguish between the different types of preclinical studies when searching for new pharmacological treatment strategies of humans, since the different types of preclinical studies, model different stages of the human condition. Overall, preclinical studies of addiction can be divided into two groups investigating either the acute rewarding aspects of psychostimulants, or the aspects of repeated exposure and relapse. We have included both types of preclinical studies in this review since both are relevant for different reasons. Pharmaceuticals targeting the direct effects of the psychostimulants might eventually lead to less consumption of them through decreased reward signalling. Preclinical studies with pharmaceuticals altering the outcome of repeated exposure or relapse of psychostimulants are relevant for the clinical aspects of withdrawal and relapse of humans suffering from psychostimulant addiction and involve animal models such as conditioned place preference, self-administration, reinstatement experiments, and behavioural sensitization (Spanagel, 2017). In recent years, more thoughts on improving preclinical research of addiction and relapse by reverse-translating successful clinical research to develop further on the existing preclinical models to test new drugs against existing ones have emerged (Venniro et al., 2020). Another interesting approach is to introduce more complexity to the pre-clinical models already in use, by for instance adding environmental differences in the setup to manipulate the vulnerability of increased drug intake of the test subjects (Fouyssac et al., 2021).

Even though the focus of this review is pharmacological treatment of psychostimulant addiction, other strategies to treat psychostimulant addiction, such as transcranial magnetic stimulation [for review see (Diana et al., 2017)], deep brain stimulation [for review see (Bina & Langevin, 2018)], psychosocial interventions (contingency management) [for review see (De Crescenzo et al., 2018)] and post-retrieval extinction paradigms [for review see (Walsh et al., 2018)] should not be overlooked.

## Targeting the dopamine system

Due to the role of dopamine in adaptive motivated behaviour, it makes sense that agonistic drugs of dopamine signalling would be highly addictive (Yuan et al., 2019). To properly understand the destructive nature of psychostimulant addiction, the understanding of the role dopamine plays on locomotion and normal reward related behaviour is essential. Natural rewards, like food and sex, lead to elevated dopamine in nucleus accumbens. Dopamine signalling is linked to both positive and negative reinforcements associated with reward-associated behaviour. The ability to make a cue-association to natural rewards is essential for survival, since evolutionarily it is important in order to both reproduce and obtain nutrients etc., (Schultz, 2006).

By directly blocking the effect of the psychostimulants themselves, decreased reward-signalling upon psychostimulant administration might eventually lead to decreased drug use. Furthermore, diminished dopamine-associated craving and tendency to relapse (Heidbreder, 2005) along with changes in frontal dopamine transmission affecting the compulsive drug intake and inhibitory control could improve treatment outcomes (Brunetti et al., 2012). However, it is important to keep in mind that any pharmaceutical affecting the acute rewarding aspects of psychostimulants might also affect the natural reward system of the brain. The experiences from treating opioid addiction with opioid receptor antagonists (i.e., naltrexone), agonists (i.e., methadone), and partial agonists (i.e., buprenorphine) have encouraged the same strategies to be approached in psychostimulant addiction (Bergman, 2008).

In this review, different approaches to targeting the dopamine system will be put forth: inhibition of the dopamine transporter with dopamine transporter (DAT) inhibitors, indirectly influencing dopamine release with psychostimulants or disulfiram, and manipulating dopamine receptors with antipsychotics or specifically targeting the D2- or D3-receptors. The results from preclinical studies examining changes in rewarding properties of psychostimulants and the preliminary results from clinical trials will be emphasized. The equivalent of decreasing rewarding properties of a drug in an animal model of addiction would be decreased self-administration, reduced conditioning (for instance in a conditioned place preference paradigm) and changes in reward threshold. In human laboratory studies, mainly changes in self-administration and impulsivity would indicate altered rewarding drug-effects (Koob et al., 2009).

### Dopamine transporter ligands and inhibitors

The dopamine transporter is of vital importance in dopamine signalling with the responsibility of clearing released dopamine from the synaptic clefts. The dopamine transporter is localized presynaptically, and following reuptake, dopamine gets packed into vesicles by the vesicle monoamine transporter (vMAT2) ready to be released again. Both cocaine and amphetamine exert their actions directly on the dopamine transporter, and therefore multiple pharmaceuticals targeting the dopamine transporter directly have been tested as a way of treating psychostimulant addiction. These pharmaceuticals are thought to function as maintenance therapy and as stabilizers of desensitized dopamine transmission during withdrawal (Jupp & Lawrence, 2010). Both DAT ligands and so-called atypical DAT-inhibitors have been investigated, and especially the atypical inhibitors seem promising, as they inhibit the DAT transporter with high affinity without leading to cocaine-like effects/symptoms (i.e., self-administration) (Reith et al., 2015). Atypical DAT inhibitors bind the transporter with characteristics differing from cocaine. For instance, the benztropine analogues bind to DAT in a manner that is distinct from that of cocaine (Schmitt et al., 2008). Uncovering the exact mechanism of action of the atypical DAT inhibitors might provide novel pharmaceutical targets, with the ability to minimize addiction symptoms, without having addictive properties themselves (Reith et al., 2015).

We will go through the most promising DAT ligands and inhibitors below. A few DAT inhibitors have already been through several clinical trials for treatment of methamphetamine addiction, some of which will be mentioned below.

#### Amphetamine and derivatives thereof

Inspired by the efficacy of agonist-based treatment of nicotine and opioid use disorders, several amphetamines have been investigated as potential maintenance treatment for cocaine use disorder [for reviews see (Howell & Negus, 2014; Negus & Henningfield, 2015)]. Amphetamine and long-acting mixed amphetamine salts have been thoroughly investigated and have shown a potential to decrease self-administration of cocaine in both rats (Chiodo et al., 2008), nonhuman primates (Negus & Mello, 2003) and humans in both a preclinical laboratory setting (Rush et al., 2010) and in clinical trials (Grabowski et al., 2001). It is believed that amphetamine (and other dopamine releasers) will only be effective to treat abuse of dopamine uptake inhibitors (like cocaine) and not be effective against other dopamine releasers, since clinical trials with amphetamine maintenance have so far failed to treat methamphetamine use disorder (Longo et al., 2010; Galloway et al., 2011; Pike et al., 2014). The great abuse liability of amphetamine and its derivatives warrant concern in relation to approval of this strategy for treatment (Negus & Henningfield, 2015), but the amount of successful human trials with long-acting amphetamine (Grabowski et al., 2001; Grabowski et al., 2004; Nuijten et al., 2016) highlights the potential of targeting the dopamine transporter as a viable target for future treatments.

#### Bupropion

Bupropion, an atypical antidepressant, has been proposed as a DAT ligand-based treatment of psychostimulant addiction and tested for psychostimulant addiction in several clinical trials. Bupropion inhibits both DAT and NET, but has no SERT activity (Stahl et al., 2004). Interestingly, bupropion has no appreciable affinity for postsynaptic receptors of neither histamine, dopamine, acetylcholine or epinephrine (Stahl et al., 2004). Bupropion has been found to decrease both acquisition (Reichel et al., 2008) and maintenance of methamphetamine self-administration in rats (Reichel et al., 2009). In addition, bupropion has been found to attenuate drug-induced reinstatement of methamphetamine seeking in rats, thus implying efficacy in relapse as well (Charntikov et al., 2018). However, since bupropion is thought to manipulate the reward system, the therapeutic has also been shown to decrease sucrose-seeking, and although not significant the treatment has shown a tendency toward inducing reinstatement of methamphetamine seeking in rats (Charntikov et al., 2018).

In human studies, bupropion has been shown to decrease subjective effects of methamphetamine as well as decrease cue-induced craving (Newton et al., 2006). A double-blind placebo-controlled study found that bupropion in combination with behavioural group therapy decreased methamphetamine intake in male low- to moderate baseline methamphetamine users independently of comorbidity (Elkashef et al., 2008). Another human study revealed that bupropion-treatment reduced the preference for intranasal cocaine over a small monetary reward (Stoops et al., 2012). The importance of proper stratification of test subjects for clinical trials is becoming clear substantiated by a human trial concluding that bupropion-treatment might only work in a very small group of people with methamphetamine addiction characterized by less than daily amphetamine use at the time of treatment (Heinzerling et al., 2014). Indeed, a pilot study on adolescents (age 14-21) concluded that bupropion-treatment did not seem to influence methamphetamine use in adolescents, interestingly showing less success in females than males (Heinzerling et al., 2013). In a recent human study with combination-treatment of oral extended-release bupropion and extended-release injectable naltrexone for treatment of methamphetamine, the bupropion group handed in more methamphetamine-negative urine samples than the placebo group despite of treatment side-effects like tremor, malaise, and hyperhidrosis (Trivedi et al., 2021).

#### Benztropine analogs

Benztropine analogs constitute a group of atypical DAT inhibitors that have been thoroughly tested in rodents but are yet to go through clinical trials (Jordan et al., 2019a). Benztropine itself does not appear to change the response to cocaine administration in humans (Penetar et al., 2006), but its ability to change cocaine craving has been tested in a clinical trial (ClinicalTrials.gov Identifier: NCT00000333). One of the more studied benztropine analogs is the atypical DAT inhibitor JHW007, which has a slow onset and a long-lasting profile (K_i_ DAT = 25 nM, K_i_ NET = 1,330 nM, K_i_ SERT = 1730 nM) (Desai et al., 2014; Jordan et al., 2019b).

The benztropine analogs AHN1-055, AHN2-005, and JHW007 dose-dependently decrease cocaine and d-methamphetamine self-administration selectively, without affecting self-administration of heroin and ketamine self-administration in rats (Hiranita et al., 2009; Hiranita et al., 2014). Moreover, AHN1-055 and JHW007 have been shown to reduce cocaine-induced locomotion without inducing motor stereotypies. Additionally, they have been shown to dose-dependently block cocaine-induced conditioned place preference, without inducing benztropine analog-induced conditioned place preference on their own (Velazquez-Sanchez et al., 2009; Velazquez-Sanchez et al., 2010). Furthermore, AHN-1055 has also been shown to lead to extinction of cocaine self-administration and reduce drug-induced reinstatement of cocaine seeking in rats (Velazquez-Sanchez et al., 2013). However, the use of benztropine analogs for psychostimulant addiction might not be as straight forward after all, since the ability of JHW007 to reduce methamphetamine self-administration might be because it enhances the reinforcing properties of the drug (Ferragud et al., 2014). Furthermore, JHW007 seems to increase certain aspects of anxiety-like behaviour in the elevated plus maze (Velazquez-Sanchez et al., 2010), which could entail a negative effect in the withdrawal stage.

#### Modafinil

Modafinil is currently used to treat narcolepsy, shift work sleep disorder and obstructive sleep apnoea. The primary mechanism of modafinil is inhibition of the dopamine transporter (Zolkowska et al., 2009; Andersen et al., 2010). However, modafinil influences both dopamine-, serotonin-, norepinephrine-, GABA-, orexin-, histamine-, and glutamate-systems in the CNS (Minzenberg & Carter, 2008). Therefore, the effects of modafinil on psychostimulant use disorder could be through an increased dopamine transmission as well as by rectifying the glutamate depletion.

Modafinil has been shown to reduce drug-induced reinstatement of cocaine seeking in rats (Mahler et al., 2014). In the same experiment, modafinil increases glutamate levels in the nucleus accumbens during chronic cocaine intake and the effect modafinil has on reinstatement can be inhibited by administration of an mGluR2/3 antagonist (LY-341495), thus supporting that albeit modafinil has broad pharmacodynamic properties, the behavioural results are likely derived from an increase in extrasynaptic glutamate (Mahler et al., 2014). Importantly, some of the additional effects of modafinil might actually prove an advantage in addiction treatment, as a cognitive enhancer (Sofuoglu et al., 2016) and as an atypical DAT inhibitor (Reith et al., 2015).

At the time of writing, 26 clinical trials of testing modafinil as treatment of different aspects of psychostimulant addiction has been performed (19 for cocaine, 8 for amphetamine) with a phase 4 trial for cocaine addiction still recruiting (ClinicalTrials.gov Identifier: NCT00439049). The clinical trials have had varying outcomes. Pre-treatment with modafinil has been shown to blunt cocaine-induced euphoria (Dackis et al., 2003) and decrease self-administration of cocaine in non-treatment seeking frequent cocaine users (Hart et al., 2008). A five-day treatment paradigm with modafinil of non-treatment seeking cocaine-dependent people reduced ratings of the “high” rated from an i.v. injection of cocaine compared to placebo treatment (Verrico et al., 2014). Other studies have shown no, or mixed effect of modafinil on psychostimulant addiction. A couple of hypotheses for the lack of effect in some of these studies have been proposed. One of them is the necessity of adherence to treatment for the modafinil to make a difference (Anderson et al., 2012). Another hypothesis is that modafinil will only work in the absence of specific comorbidities. For instance, post-hoc analysis of one of the bigger clinical trials initially showing no effect of modafinil on cocaine-dependence, showed that there was indeed a significant effect, if the subgroup of patients with comorbid alcohol abuse were removed from the analysis (Anderson et al., 2009). Shortly after that, a second study recruiting cocaine-dependent subjects specifically without alcohol-dependence confirmed the same finding (Kampman et al., 2015).

Isomers of modafinil are of interest too, since both R-modafinil and S-modafinil bind the dopamine transporter and inhibit dopamine uptake with different profiles than cocaine, making them interesting candidates for substitute therapy of psychostimulant use disorders (Loland et al., 2012).

### Targeting dopamine receptors

Upon release into the synaptic cleft, dopamine exerts its signalling function by binding to G-protein coupled dopamine receptors (GPCRs), classically divided into the stimulatory D1 class receptors (D1- and D5-receptors) and the inhibitory D2 class receptors (D2-, D3-, and D4-receptors) based on the G-protein coupling and differences in their pharmacological profiles. Differences in expression patterns and G-protein coupling of the dopamine receptors ensure the necessary feedback loops to preserve proper levels of dopamine signalling and both D1- and D2-receptors are necessary for the reinforcing effect of drugs of abuse (Woolverton et al., 1984; Self & Stein, 1992; Weed & Woolverton, 1995; Ranaldi et al., 2001; Volkow et al., 2002).

#### Antipsychotics targeting D2-receptors

Antipsychotics can be divided into typical antipsychotics (e.g., haloperidol) and atypical antipsychotics (e.g., clozapine). Antipsychotics are used to treat mental disorders such as schizophrenia and bipolar disorder, both of which show significant comorbidity with substance use disorder (Brown et al., 2003; Hanley & Kenna, 2008). Rodent studies have shown that the typical antipsychotics lead to increased cocaine self-administration, and humans treated with typical antipsychotics, while suffering from cocaine addiction, have been reported to receive higher doses of the antipsychotics than their none cocaine-addicted counterparts (Seibyl et al., 1993; Brown et al., 2003). However, to varying degrees, the atypical antipsychotics show a more promising mechanism in relation to treating cocaine use disorder than their typical counterparts, and some of these atypical antipsychotics, (e.g., the partial agonist aripiprazole and the antagonist quetiapine), have been investigated as potential therapeutics in animal models as well as humans with varying success.

##### Quetiapine

Quetiapine is a weak antagonist of the D2-receptor and a strong antagonist of the 5-HT2A-receptor. Additionally, it has antagonistic properties towards H1-, α1- and 2-, 5-HT1A-, and 5-HT3A-receptors (Rowe, 2007). Quetiapine is effectively used to treat schizophrenia and bipolar disorder (Hanley & Kenna, 2008). Initial research of quetiapine as a treatment option for psychostimulant addiction derives from reports of patients treated with an antipsychotic for a mental illness while dependent on cocaine and/or amphetamine reporting reductions in psychostimulant use (Brown et al., 2002; Brown et al., 2003; Hanley & Kenna, 2008). In rats, quetiapine has been shown to prevent cocaine-induced enhancement of brain-stimulated reward (Gallo et al., 2010). In a self-administration study on adult rhesus monkeys, acute quetiapine decreased cocaine choice in 4 out of 7 monkeys, but tolerance for quetiapine developed within 14–16 days diminishing the relevance of quetiapine as a monotherapy of cocaine use disorder (Brutcher & Nader, 2015). Furthermore, quetiapine failed to decrease self-reported cocaine use, drug-urine concentrations, self-reported money spent on cocaine and cocaine craving in a randomized, double-blinded, placebo-controlled study of 60 cocaine users compared to placebo (Tapp et al., 2015) and quetiapine has been shown to worsen anhedonia during methamphetamine-withdrawal in rats (Zhornitsky et al., 2010). Finally, emerging reports on quetiapine abuse is a valid concern for the clinical use of quetiapine (Hanley and Kenna, 2008).

##### Aripiprazole

Aripiprazole is a partial agonist of the D2- and 5-HT1A-receptors, and an antagonist of the 5-HT2A-receptor. Furthermore, it has affinity for D3-, D4-, 5-HT2C-, 5-HT7, α1-adrenergic-, and histaminergic-receptors and the 5-HT reuptake site along with an anti-inflammatory effect *via* inhibition of microglial activation (Brunetti et al., 2012). In rats, aripiprazole has been shown to decrease self-administration of methamphetamine in a progressive-ratio as well as a fixed-ratio schedule, and this was seen for rats with short as well as long access to methamphetamine (Wee et al., 2007). In humans, aripiprazole has been shown to attenuate some of the positive subjective effects of amphetamines but without affecting their drug-discriminative performance (Stoops et al., 2006). In another human study, long-term aripiprazole administration was found to increase the use of smoked cocaine in humans, possibly to circumvent the blunted effect of the drug, which would indicate that using aripiprazole to treat individuals with cocaine addiction that are still administering cocaine will be unsuccessful (Haney et al., 2011). However, rat-studies have shown that both acute and chronic aripiprazole decrease cue- and cocaine-induced reinstatement (Feltenstein et al., 2007; Feltenstein et al., 2009). The exact mechanism of action resulting in this behavioural change is still largely unknown and because of the broad pharmacodynamics of aripiprazole both D2-antagonism, 5-HT1A receptor-interaction and regulation of glutamatergic transmission in the prefrontal cortex have been proposed (Feltenstein et al., 2009). In 2013, two human studies were published testing the possibility of treating methamphetamine addiction with aripiprazole (Coffin et al., 2013; Sulaiman et al., 2013). One of the studies concluded that aripiprazole did not reduce methamphetamine use among actively using dependent adults (Coffin et al., 2013). The other study concluded that while aripiprazole was unable to help maintaining abstinence of the individuals addicted to methamphetamine, it did reduce the severity of psychotic symptoms (Sulaiman et al., 2013).

##### Clozapine

The atypical antipsychotic clozapine was used for treatment of schizophrenia in Europe for the first time in 1971, but has since had a turbulent history of withdrawal from the market due to fatal side-effects and reoccurrence onto the market as a treatment strategy to reduce recurrent suicidal behaviour of schizophrenia patients and to treat treatment-resistant schizophrenia (Warnez & Alessi-Severini, 2014; Chiappini et al., 2020). In this review, we have chosen to place clozapine as a D2-receptor antagonist, even though the combination of pharmacological effects of clozapine is quite unique interacting with dopamine-, adrenergic-, glutamatergic-, histaminic-, and muscarinic-receptors (Chiappini et al., 2020).

In rats, clozapine has been shown to prevent the cocaine-induced increase in nucleus accumbens dopamine release as well as cocaine-induced hyperlocomotion after acute administration of cocaine (Broderick et al., 2004). In other studies, on both mice and rats, clozapine has been shown to also prevent sensitized hyperlocomotion induced by cocaine (Filip et al., 2003; Cha & Kang, 2014) or amphetamine (Meng et al., 1998). Additionally, clozapine decreases cocaine self-administration in rats (Roberts & Vickers, 1984). In humans, patients with co-occurring schizophrenia and substance use disorders (including cocaine use disorder), on clozapine-treatment, show a significant decrease in relapse of drug abuse at both 6-months, 1-year, and 2-year follow up (Brunette et al., 2006). A comprehensive review on possible withdrawal and abuse issues from clozapine use came out recently, and confirmed that abrupt discontinuation of clozapine can lead to withdrawal symptoms and that high-dose intake of clozapine can lead to severe and fatal consequences thereby solidifying the importance of proper introduction by health care professionals before prescribing the pharmaceutical (Chiappini et al., 2020).

#### D3-receptor antagonists

D2-receptors are found in all the major brain areas receiving dopaminergic projections, whereas the mRNA of D3-receptors is mainly found in telencephalic areas receiving inputs from the A10 cell group (Bouthenet et al., 1991). The more restricted localization of D3-receptors compared to D2-receptors has made them a focus of interest for addiction treatment (Le Foll et al., 2014). Human PET studies have revealed an increase in D3-receptor expression in psychostimulant users, encouraging studies into highly specific D3-receptor antagonists (Le Foll et al., 2014; Matuskey et al., 2014). In rats, the selective D3-receptor antagonist SB-277011A has been found to dose-dependently attenuate both cocaine-induced conditioned place preference and cocaine-induced reinstatement of cocaine seeking behaviour (Vorel et al., 2002). The same study also showed the ability of SB-277011A to prevent the rewarding effects of cocaine, by blocking enhancement of brain stimulation-induced reward by cocaine in rats signifying the involvement of D3 receptors in the rewarding properties of cocaine (Vorel et al., 2002). In addition, SB-277011A has been found to reduce both cue- and stress-induced reinstatement of cocaine seeking in rats (Xi et al., 2004; Gilbert et al., 2005). Furthermore, SB-277011A has been shown to decrease self-administration of cocaine in low doses, and to lower the breakpoint of cocaine-seeking in a progressive-ratio schedule when the work-demand for reward was increased in rat self-administration studies (Xi et al., 2005). Another selective D3-antagonist, SR21502, has been shown to significantly accelerate extinction of cocaine-seeking in a cocaine-conditioned place preference paradigm without showing any abilities to induce conditioned place preference on its own (Galaj et al., 2016). Several other D3-receptor antagonists have been investigated and have shown promising results, but a potential for cardiovascular side effects e.g., increased blood pressure (Jordan et al., 2019a) along with lack of efficacy in low cost self-administration paradigms (Di Ciano et al., 2003; Xi et al., 2005) have limited further progress of the abovementioned antagonists. These issues have instead incited development of compounds with high affinity and selectivity (e.g., R-VK4-116 and R-VK4-40), which does not give rise to cardiovascular effects (Jordan et al., 2019b), along with investigations into efficacy of partial D3 receptor agonists such as the atypical antipsychotic cariprazine, which have shown to decrease self-administration of cocaine and attenuate cue-induced reinstatement of cocaine seeking in rats (Roman et al., 2013) [for reviews see (Newman et al., 2021; Newman et al., 2022)]. Although the role of D3-receptors in addiction is not yet fully understood, studies targeting these receptors in addiction treatment so far look promising, and it will be interesting to follow the development of this field in years to come.

### Dopamine metabolism

Psychostimulants manipulate the dopamine system directly by increasing dopamine levels in the synaptic cleft through targeting of the dopamine transporter. The treatments discussed so far, all target different steps of the direct dopamine signalling but indirect manipulation of the dopamine system has also been investigated for treatment of psychostimulant addiction.

#### Disulfiram

A way to indirectly manipulate dopamine signalling is by targeting the dopamine metabolism with pharmaceuticals like disulfiram. Disulfiram is a well-known treatment for alcohol-dependence, which is currently being investigated as a potential pharmaceutical for treating cocaine dependence (Charpeaud et al., 2011). Studies have shown that besides its effect on aldehyde dehydrogenase, disulfiram also inhibits dopamine beta-hydroxylase (the enzyme responsible for converting dopamine to norepinephrine). This inhibition occurs by diethyldithiocarbamate, the reduction product of disulfiram, through chelation of the copper ions that are important cofactors for the activity of the enzyme (Goldstein et al., 1964). The exact consequences of this is still largely unknown, but an inhibition of psychostimulant-induced dopamine release through decreased noradrenaline has been suggested, as the positive effects of disulfiram on cocaine-dependence cannot be explained by an increased baseline dopamine concentration alone (Gaval-Cruz & Weinshenker, 2009).

In a human study, participants receiving disulfiram and cognitive behavioural therapy were found to have lower cocaine use, than those who received cognitive behavioural therapy and placebo. Furthermore, this effect was larger in participants who were not alcohol dependent or were abstinent during the trial, thus indicating that the effect was not because of reduced alcohol use (Carroll et al., 2004). Another human study, reported that the “high” resulting from i.v. administration of cocaine was diminished upon acute disulfiram treatment (Baker et al., 2007). A third study revealed significantly longer cocaine abstinence in opioid and cocaine-dependent patients on disulfiram compared to placebo (George et al., 2000). Interestingly, a single human study has looked at gender difference of treating individuals with cocaine addiction with disulfiram indicating that this treatment might only be useful in males (Nich et al., 2004).

Though these results are promising, several other randomized controlled trials have been conducted investigating an effect of disulfiram on cocaine dependency with varying results. Many of the studies have had low numbers, high heterogeneity of abuse or safety issues, possibly due to a cocaine-disulfiram interaction (Pani et al., 2010) but interestingly, some studies indicate that in addition to gender, different genotypes of the dopamine beta-hydroxylase might influence the effect of disulfiram on cocaine addiction (Kosten et al., 2013). Perhaps further research in this area or greater stratification of the clinical trials could provide additional evidence of treatment efficacy.

### Summary of pharmacologic approaches targeting the dopamine system

The rationale for interfering with the dopaminergic transmission in addiction disorders is in many ways straightforward given that psychostimulant addiction is considered a disorder of the dopamine driven reward system. Consequently, occupying the binding site for psychostimulants on the dopamine transporter, targeting the downstream receptors or modulating the dopamine metabolism is likely to reduce the rewarding effects of psychostimulants and thus might alter habit formation. Using dopaminergic medications to treat stimulant use disorders is resembling replacement therapy, which is an already established strategy successful in the treatment of nicotine and opioid dependence (Frishman, 2007; Hays & Ebbert, 2008; Kampman & Jarvis, 2015). For an overview of all clinically tested drugs affecting the dopamine system mentioned in this review, see table 1.

**TABLE 1:** Human studies with pharmacological targeting of the dopamine system to treat psychostimulant addiction.

	Drug	References	Participants	Drug concentration	Results
Dopamine transporter	Bupropion	Newton et al. (2006)	20 (10 bupropion, 10 placebo) non-treatment seeking adult methamphetamine users	Placebo or 150 mg extended-release bupropion	Bupropion decreased subjective effect and cue-induced craving of methamphetamine
		Elkashew et al. (2008)	151 treatment-seeking methamphetamine users of both genders in a double-blind placebo-controlled study with 12 weeks treatment and 30 days follow up	Placebo or sustained-release bupropion 150 mg twice daily + behavioural group therapy	Bupropion in combination with behavioural group therapy has a significant effect in male low- to moderate baseline amphetamine users independently of comorbidity
		Stoops et al. (2012)	8 cocaine-using adults	Pre-treatment with 0, 100 or 200 mg oral immediate-release bupropion	Bupropion reduced the preference for intranasal cocaine over a small monetary reward
		Heinzerling et al. (2013)	19 adolescent (age 14–21) methamphetamine-users (randomly divided into Bupropion vs. placebo groups)	Placebo or 150 mg sustained-release bupropion, twice daily for 8 weeks	Bupropion has no effect on methamphetamine use of adolescent females with a trend for greater retention among males
		Heinzerling et al. (2014)	84 (41 bupropion, 43 placebo) methamphetamine-dependent volunteers	Placebo or 150 mg bupropion, twice daily	Bupropion-treatment might only work in methamphetamine addicts with less than daily amphetamine use at the time of treatment and proper medication adherence throughout the treatment window
		Trivedi et al. (2021)	403 adults with moderate to severe methamphetamine use disorder divided into different ratios of either bupropion:naltrexone or placebo treatment	Placebo or up to 450 mg oral extended-release bupropion per day and 380 mg extended-release injectable naltrexone every 3 weeks	Combination treatment of bupropion and naltrexone resulted in an increased amount of methamphetamine-negative urine samples compared to placebo group
	Modafinil	Dackis et al. (2003)	10 male cocaine-dependent individuals in a placebo-controlled trial	Pretreatment with placebo, 200 mg, or 400 mg modafinil for 4 days prior of 30 mg i.v. cocaine	Modafinil pre-treatment blunts cocaine-induced euphoria
		Hart et al. (2008)	8 (7 male, 1 female) smoked cocaine-dependent individuals using cocaine 4 days/week in a placebo-controlled trial	Placebo, 200 mg, or 400 mg oral modafinil/day in a within-subject, alternating in-patient/outpatient 48-day study	Modafinil attenuated self-administration, subjective-effect ratings and cardiovascular measures of the effect of cocaine
		Anderson et al. (2009)	210 (72 placebo, 69 200 mg modafinil, 69 400 mg modafinil) treatment-seeking cocaine-dependent individuals	Placebo, 200 mg, or 400 mg modafinil/day on awakening for 12 weeks. Followed by a 4 week follow up	Only 200 mg modafinil had a significant effect on maximum number of consecutive days without cocaine use and a reduction in craving. The subgroup of participants without a history of alcohol dependence had an increased weekly percentage of days without cocaine use for both tested modafinil concentrations
		Anderson et al. (2012)	210 (68 placebo, 72 200 mg modafinil, 70 400 mg modafinil) treatment-seeking methamphetamine-dependent individuals	Placebo, 200 mg, or 400 mg modafinil/day on awakening for 12 weeks. Followed by a 4 week follow up	Overall, no effect of modafinil was found. However, a significant corelation between urine-levels of modafinil and maximum duration of abstinence was found
		Verrico et al. (2014)	64 (16 placebo, 16 modafinil, 17 escitalopram, 15 escitalopram + modafinil)	Placebo, 200 mg modafinil, 20 mg escitalopram or 200 mg modafinil plus 20 mg escitalopram per day for 5 days	Modafinil significantly attenuated positive subjective effects of cocaine independent of escitalopram
		Kampman et al. (2015)	94 cocaine-dependent individuals in placebo-controlled trial	Placebo or 300 mg modafinil daily for 8 weeks	Modafinil-treated subjects were significantly more likely to be abstinent of cocaine use during the last 3 weeks of the 8-week trial compared to the placebo group with low levels of craving
		Haney et al. (2011)	8 male cocaine-dependent individuals	Placebo or 15 mg capsulated aripiprazole/day	Aripiprazole decreased ratings of cocaine quality, craving and effect compared to placebo, but increased use of smoked cocaine
		Coffin et al. (2013)	90 actively using methamphetamine-dependent adults	5 mg aripiprazole/day for 1 week, then 10 mg aripiprazole/day for 1 week, then 20 mg aripiprazole/day for 10 weeks	Aripiprazole was unable to reduce methamphetamine use among actively using dependent adults
		Sulaiman et al. (2013)	37 (19 aripiprazole, 18 placebo) methamphetamine-dependent humans	Placebo or 5–10 mg aripiprazole/day for 8 weeks	Aripiprazole was unable to help maintaining abstinence of methamphetamine users but reduced the severity of psychotic symptoms
	Amphetamines	Grabowski et al. (2001)	128 cocaine-dependent subjects	12-week randomized, double-blind, placebo-controlled trial with 0, 15 mg, or 30 mg sustained-release dextroamphetamine for 5 weeks followed by 0, 30 mg, or 60 mg sustained-release dextroamphetamine for 7 weeks. Incl. 1 behavioural therapy session/week	Twice weekly urine samples revealed that the low dose of sustained-release dextroamphetamine resulted in improved retention
		Grabowski et al. (2004)	120 cocaine-and heroin-dependent subjects of 18–50 years of age	26 weeks, randomized, double-blind and placebo-controlled trial with stable methadone throughout the study (1.1 mg/kg) and 0, 15 mg, or 30 mg sustained-release dextroamphetamine for 4 weeks followed by 0, 30 mg, or 60 mg sustained-release dextroamphetamine for 20 weeks. Incl. 1 behavioural therapy session/week	Twice weekly urine samples as well as self-report measures revealed a significant reduction in cocaine use for the highest dose of sustained- release dextroamphetamine with reduced heroin use in all groups with a trend towards higher reduction in the high dose sustained-release dextroamphetamine group
		Rush et al. (2010)	9 non-treatment seeking cocaine using adults of 28–50 years. 7 male, 2 female	Within-subjects placebo-controlled study with 0 mg or 40 mg/day sustained-release d-amphetamine	Blinded choice test between 4 mg, 10 mg, 20 mg, or 30 mg cocaine. 20 mg cocaine were chosen less while on 40 mg sustained-release d-amphetamine compared to 0 mg sustained-release d-amphetamine
		Nuijten et al. (2016)	Randomised, double-blind, placebo-controlled trial of 73 treatment-refractory crack-cocaine users	12 weeks of daily, supervised prescription of 60 mg/day oral sustained-release dexamphetamine or placebo in addition to co-prescribed methadone and diacetylmorphine	Sustained-release dexamphetamine treatment resulted in significantly fewer days of cocaine use than placebo treatment
		Longo et al. (2010)	Randomized, double-blind, placebo-controlled trial with 49 methamphetamine-dependent drug users	Up to 110 mg/day sustained-release dexamphetamine (*n* = 23) or placebo (*n* = 26) for a maximum of 12 weeks, with gradual reduction of the study medication over an additional 4 weeks	Significant decrease in methamphetamine concentration for both groups with the degree of methamphetamine-dependence being significantly lower in the dexamphetamine group at follow-up
		Galloway et al. (2011)	Randomized, double-blind, placebo-controlled trial with 60 methamphetamine-dependent drug users age 18-50 of both genders	60 mg/day slow release d-amphetamine or placebo daily for 8 weeks (administered as a single dose on day 1 and two doses on subsequent days) and 8 psychotherapy sessions	No significant difference in amount of methamphetamine-positive urine samples, but the treatment group reported less craving and withdrawal
		Pike et al. (2014)	Within-subjects, placebo-controlled, double-blind study with 8 non-treatment seeking males, age 24–52	0 or 40 mg/day of sustained release d-amphetamine administered over two doses per day	d-amphetamine did not significantly reduce self-administration of methamphetamine
Dopamine receptors	Quetiapine	Tapp et al. (2015)	12-week, prospective, double-blind, randomized, placebo-controlled study with 68 subjects	29 subjects were titrated into receiving up to a target dose of 400 mg/day of quetiapine with a weekly cognitive-behavioural therapy group session	No effects of quetiapine for treatment of cocaine-dependence
	Aripiprazole	Stoops et al. (2006)	20–33 experimental sessions with 6 healthy adults (3 males, 3 females) in a placebo-controlled study	0, 2.5, 5, 10, and 15 mg of d-amphetamine and 0 and 10 mg of aripiprazole	10 mg of aripiprazole did not attenuate the discriminative-stimulus effects of d-amphetamine but reduced some of the positive subject-rated effects produced by d-amphetamine
		Haney et al. (2011)	A 42-day, within-subject, human laboratory study with 8 nontreatment-seeking, cocaine-dependent males	Placebo or 15 mg/day aripiprazole	Aripiprazole significantly increased cocaine (12, 25 mg) self-administration. Following repeated cocaine (50 mg) self-administration, aripiprazole decreased ratings of cocaine quality and craving compared to placebo
		Coffin et al. (2013)	90 actively using, methamphetamine-dependent males and females ages 18–60 in a double-blind, placebo-controlled, randomized trial	12 weeks of aripiprazole (week1: 5 mg/day, week 2: 10 mg/day, week 3–12: 20 mg/day) or placebo, with a 3-month follow-up and weekly 30-min substance abuse counseling	Compared with placebo, aripiprazole did not reduce methamphetamine use significantly among actively using, dependent adults
		Sulaiman et al. (2013)	Double-blind study of 37 methamphetamine-dependent patients with history of psychosis	5–10 mg/day aripiprazole or placebo for 8 weeks with follow-up on day 7, 14, 28, 42 day 56 after enrolment	Aripiprazole reduced the severity of psychotic symptoms but was no more effective than placebo in maintaining abstinence from methamphetamine use
	Clozapine	Brunette et al. (2006)	169 males and females with comorbidity of substance use diorder and schizophrenia or schizoaffective disorder	Mean clozapine dose was 417 mg (SD = 166) 3 months prior to the interview. Mean clozapine dose was 484 mg (SD = 157) at 1 year after remission	Patients using clozapine at the first 6-month period of substance abuse remission (*n* = 25) were less likely to relapse over the next year
Dopamine metabolism	Disulfiram	George et al. (2000)	15 (8 disulfiram, 7 placebo) buprenorphine-maintained opioid-and cocaine-dependent individuals	Placebo or 250 mg/day disulfiram for 12 weeks	Disulfiram-group was cocaine-abstinent significantly longer than the placebo group with more cocaine-negative urine tests
		Carroll et al. (2004)	121 (treated with either disulfiram or placebo) cocaine-dependent individuals	Placebo or 250 mg/day disulfiram for 12 weeks	Individuals treated with disulfiram had a significantly reduced cocaine intake compared to the placebo group, with highest effects on individuals with no alcohol intake
		Nich et al. (2004)	67 cocaine-dependent individuals on methadone maintenance. Disulfiram vs. placebo	Placebo or 250 mg disulfiram twice weekly for 12 weeks	Disulfiram treatment significantly decreased cocaine use in male participants only
		Baker et al. (2007)	Non-treatment seeking, cocaine-dependent, volunteers. Disulfiram vs. placebo	Placebo, 62.5 mg, or 250 mg disulfiram per day for 6 days	Disulfiram groups showed decreased cocaine clearance, cocaine ‘high’ and cocaine ‘rush’
		Kosten et al. (2013)	74 (34 disulfiram, 40 placebo) cocaine- and opioid co-dependent subjects on 2 weeks methadone treatment	Placebo or 250 mg disulfiram twice weekly for 10 weeks	Disulfiram treatment reduced cocaine-positive urine samples dependent on dopamine beta-hydroxylase genotype of test subjects

DAT inhibitors including Bupropion and novel benztropine analogs show promising preclinical results including beneficial effects on reward and habit formation induced by psychostimulants, but also in relation to drug-craving specifically as assessed by reinstatement. Clinical findings confirm suppression of drug-reward in humans, and Bupropion has even demonstrated moderate efficacy in reducing drug use under certain conditions. Despite their clear effects in preclinical studies, antipsychotics with primary effect on D2 receptors, have generally shown disappointing results in clinical trials except their potential for reducing drug intake and craving in patients suffering from schizophrenia or bipolar disorder. Recently, the more localized expression of D3 receptors has fuelled promising development of D3 specific antagonist but they have so far not been tested clinically. Finally, disulfiram, which inhibits dopamine beta-hydroxylase, has shown effect on drug perception, use and abstinence, but outcome is dependent on gender of the subjects and genotype variation of dopamine beta-hydroxylase. Importantly, use is possibly compromised by cocaine-disulfiram interaction.

Taken together, the strong preclinical data, and several successful clinical trials, although with modest efficacy, highlights the feasibility and potential of targeting the dopamine system to treat psychostimulant addiction. Importantly, the most relevant compounds can indeed reduce not only drug perception but also drug intake. We speculate that by circumventing large dopamine fluctuations, such drugs might be beneficial. Likely, better insight into the dopamine dynamics and the related behaviour will emerge in the wake of novel fluorescence-based dopamine sensors with high temporal resolution (Patriarchi et al., 2018; Sun et al., 2018).

## Targeting the glutamate system

Glutamate and GABA receptors are the mediators of most synaptic transmission and both types (incl. all subtypes) are involved in the induction, expression, and modulation of certain types of synaptic plasticity. Glutamate activates three main classes of ionotropic glutamate receptors named after their selective agonists; alpha-amino-3-hydroxy-5-methyl-4-isoxazole propionic acid (AMPA), N-methyl-D-aspartate (NMDA), and kainate as well as a family of G-protein coupled metabotropic glutamate receptors (mGluRs). GABA activates two main classes of receptors; the ionotropic GABA-A receptors and the G-protein coupled metabotropic GABA-B receptors (Collingridge et al., 2004). The dynamic levels of AMPA receptors at the synapses lead to plastic changes in synaptic function and are thought to underlie information storage in the brain by modifying connectivity between neurons and neuronal circuits. Therefore, AMPA receptors have been heavily studied and understanding the life cycles of AMPA receptors is fundamental to the understanding of synaptic plasticity.

Glutamate transmission, especially in the nucleus accumbens, changes radically in response to chronic psychostimulant addiction. Transmitter release, receptor expression, receptor trafficking, reuptake, and intracellular signalling all change dramatically with different adaptations in different brain areas, in different stages of addiction, and experimental settings (Schmidt & Pierce, 2010; Luscher & Malenka, 2011).

### Alpha-amino-3-hydroxy-5-methyl-4-isoxazole propionic acid receptor regulation in memory and addiction

AMPA receptors are a subtype of glutamate receptors mediating rapid excitatory neurotransmission in the CNS. They are ligand-gated ion channels and are the main mediators of excitatory neurotransmission in the brain. AMPA receptors are homo- or hetero-tetramers consisting of the four basic units, GluA1-4, undergoing different splicing and RNA editing and combining in different stoichiometries to form sodium/potassium and in some cases also calcium permeable cationic channels of different functional properties (Hollmann & Heinemann, 1994; Rosenmund et al., 1998; Shepherd & Huganir, 2007). The transmembrane and extracellular parts of the different subunits are very similar, but they vary in their intracellular cytoplasmic C-terminal part (Shepherd & Huganir, 2007). Regulation of the number and type of AMPA receptors on the plasma membrane is divided into long-term potentiation (LTP) and long-term depression (LTD). Under basal conditions AMPA receptors are constitutively trafficking with repeated cycles of endocytosis and reinsertion allowing for an effective and fast control of receptor density (Nishimune et al., 1998; Luscher et al., 1999; Carroll et al., 2001; Malinow & Malenka, 2002; Bredt & Nicoll, 2003; Hanley, 2008). AMPA receptor trafficking is a fundamental mechanism for regulating synaptic strength, underlying mechanisms involved in learning and memory. The interaction of the GluA1/GluA2 subunits of AMPA receptors with several different proteins affects trafficking of AMPA receptors.

In response to chronic drug abuse changes in both LTD and LTP have been shown both in the VTA and in the glutamatergic corticostriatal projections terminating on the medium spiny neurons (MSNs) of the shell and core of the nucleus accumbens through changes in NMDA and AMPA receptor transmission. This bidirectional plasticity is seen during different stages of addiction and in different experimental settings (Luscher & Malenka, 2011; Quintero, 2013; Mulholland et al., 2016) and with slight differences between different psychostimulants (Wolf & Ferrario, 2010). For instance, repeated cocaine administration leads to LTD in the nucleus accumbens, whereas subsequent withdrawal leads to insertion of LTP involving calcium-permeable AMPARs to mediate drug-craving (Thomas et al., 2001; Kourrich et al., 2007; Conrad et al., 2008). In general, an increase in AMPA currents, both in the nucleus accumbens (especially the D1 expressing MSNs), the orbitofrontal cortex, and the prefrontal cortex seem to be principal during extended withdrawal and underlie some of the vulnerability to relapse seen in psychostimulant addiction (Mulholland et al., 2016). Furthermore, LTD and LTP in the nucleus accumbens are pathologically inhibited by chronic cocaine self-administration, thus explaining an increased rigidity in adaptive behaviour (Kalivas & Volkow, 2011). Modulation of AMPAR signalling or reversal of the complex changes in LTP and LTD seen in addiction therefore seems like a promising target for therapeutics.

#### Direct alpha-amino-3-hydroxy-5-methyl-4-isoxazole propionic acid receptor manipulation

In rats, the AMPA-antagonist LY293558 has been shown to inhibit reinstatement of cocaine seeking after a drug-related cue when infused in the nucleus accumbens core, but not shell (Di Ciano & Everitt, 2001). Another, AMPA receptor antagonist, the anticonvulsant topiramate has been investigated more thoroughly. Of note, topiramate also inhibits voltage gated sodium and calcium channels, as well as GABA-A receptors. In humans, a pilot study with topiramate-treatment revealed an increased success rate of abstinence in patients with cocaine dependence (Kampman et al., 2004). A systematic review and meta-analysis from 2016 concluded from a total of five studies, that even though topiramate-treatment does not improve treatment retention, it was able to extend continuous cocaine abstinence in two of the five studies and significantly reduced craving in one study (Singh et al., 2016).

#### Indirect alpha-amino-3-hydroxy-5-methyl-4-isoxazole propionic acid receptor manipulation

An alternative strategy to targeting the AMPA receptor signalling directly with antagonists, is to target their subunit composition and expression at the surface. This can be done by targeting several different AMPA receptor interaction partners. In this review we have chosen to focus on the C-terminal binding partner of GluA2-containing AMPA receptors, PICK1. The scaffolding protein PICK1 is thought to influence both internalization and insertion of GluA2-containing AMPA receptors in the shell of the nucleus accumbens (Schmidt & Pierce, 2010) and the upregulation of AMPA receptors lacking the GluA2-subunit seems to depend on the downregulation of GluA2-containing AMPA receptors (Famous et al., 2008). During extinction of cocaine self-administration, the levels of PICK1 decreases in the dorsolateral striatum, while total PICK1 expression increases in the nucleus accumbens shell of rats (Ghasemzadeh et al., 2009). PICK1 knockout mice show reduced self-administration of cocaine along with increased striatal dopamine, suggesting an importance of PICK1 in regulating the dopamine homeostasis in addiction (Jensen et al., 2018). Additionally, it has been shown in mice that disrupting the interaction between GluA2-containing AMPA receptors and PICK1 with a cell-permeable peptide (Pep2-EVKI) in both the core and the shell of the nucleus accumbens attenuates drug-induced reinstatement (Famous et al., 2008) and a decrease in cocaine-induced reinstatement has also been seen in rats using a high-affinity PICK1-inhibitor (Tat-P4-(DATC5)_2_) administered systemically (Turner et al., 2020). Another peptide (Tat-GluA2(3Y) likewise regulates AMPARs surface expression, by blocking the BRAG2/Arf6-mediated endocytosis following tyrosine phosphorylation of the GluA2 C-terminus and has been shown to prevent behavioural sensitization to amphetamine in rats (Brebner et al., 2005). So far, there are no human studies using this approach to treat psychostimulant addiction.

### Metabotropic glutamate receptors

The metabotropic glutamate receptors are divided into three groups depending on their pharmacology and signal transduction. Group I receptors (mGluR1/5) are G_q_-coupled and interacts with Homer-proteins and are typically located post-synaptically. Group II receptors (mGluR2/3) are G_i/o_-coupled and located both pre- and post-synaptically. Group III receptors (mGluR4 and mGluR6-8) are also G_i/o_-coupled but predominantly located pre-synaptically (Ferraguti & Shigemoto, 2006). It has been found that mGluRs, particularly in the nucleus accumbens, influence brain reward function, regulate drug self-administration, and conditioning of drug-related stimuli (Kenny & Markou, 2004; Li & Markou, 2015). mGluRs therefore seem promising candidates for addiction treatment, although they are still early in development (Olive, 2009).

#### mGluR1/5 modulation

DHPG (mGluR1 and mGluR5 agonist), Ro67-7476 (selective mGluR1 positive allosteric modulator) and SYN119 (selective positive allosteric modulator of mGluR1) all have the ability to decrease cue-induced cocaine seeking when administered directly into the nucleus accumbens of rats following prolonged withdrawal of cocaine self-administration (Loweth et al., 2014). SYN119 has been shown to attenuate cocaine as well as methamphetamine craving in rats after systemic administration (Loweth et al., 2014; Scheyer et al., 2016; Caprioli et al., 2018). In squirrel monkeys, JNJ16259685 (selective mGluR1 agonist) attenuates self-administration, drug-discrimination and reinstatement of cocaine and methamphetamine, and in rats JNJ16259685 has been shown to attenuate drug context-induced reinstatement of cocaine seeking (Xie et al., 2010; Achat-Mendes et al., 2012).

The negative allosteric modulators of mGluR5, MTEP, and MPEP, have been found to decrease cocaine-induced reinstatement in rats and the same study found that MTEP also diminished cue-induced reinstatement of cocaine (Kumaresan et al., 2009). Despite their ability to reduce cocaine self-administration, neither MTEP nor MPEP will likely be used to treat humans suffering from psychostimulant addiction due to extensive off-target side-effects (e.g., hallucinations and interaction with hepatic enzyme CYP1A2). However, they demonstrate a potential for negative allosteric modulators of mGluR5 as future therapeutics (Mihov & Hasler, 2016).

#### mGluR2/3 modulation

The mGluR2/3 agonist LY379268 has shown decreased reinstatement of cue-induced self-administration of cocaine in rats (Baptista et al., 2004). Administration of LY379268 has also been shown to decrease cue- and drug priming-induced reinstatement of cocaine self-administration in nonhuman primates (Adewale et al., 2006; Justinova et al., 2016) and incubation of cocaine or methamphetamine self-administration in rats (Liechti et al., 2007; Crawford et al., 2013).

BINA, a positive allosteric modulator of the mGluR2, has been shown to attenuate self-administration and increase intracranial self-stimulation threshold of cocaine in rats. Furthermore, BINA also reduced cue-induced reinstatement of cocaine (Jin et al., 2010). Another selective positive allosteric modulator of mGluR2, AZD8529, has been shown to decrease incubation of methamphetamine craving in rats. When given the choice between food and methamphetamine after prolonged voluntary abstinence, the rats chose the palatable food (Caprioli et al., 2015), however, clinical trials have only been carried out for smoking cessation.

#### mGluR7 modulation

Systemic administration of AMN082, the first selective agonist for mGluR7, (Mitsukawa et al., 2005), has been shown to decrease cocaine self-administration in both a fixed and progressive ratio schedule in rats without affecting sucrose intake (Li et al., 2009). Effect on cocaine self-administration, however, is less pronounced than for nicotine self-administration (Li & Markou, 2015). Moreover, systemic and intra-accumbens administration of AMN082 (10 mg/kg) attenuated cocaine priming induced reinstatement of cocaine seeking behaviour in rats, but also to some extent sucrose-induced reinstatement of sucrose seeking (20 mg/kg) (Li & Markou, 2015).

#### Modulation of glutamate transport

The glutamate transporter GLT-1 (EAAT2 in humans) is an influential regulator of extracellular glutamate. It is primarily an astroglial transporter localized near the synaptic clefts where it eliminates glutamate from the extracellular space (Kalivas, 2009). During self-administration of cocaine, GLT-1 is downregulated in the nucleus accumbens, and this seems to greatly contribute to the heavily increased synaptic glutamate levels during reinstatement (Quintero, 2013).

#### N-acetylcysteine

N-acetylcysteine, a prodrug of cystine commonly used to treat paracetamol poisoning, seems a promising candidate for treatment of psychostimulant addiction (Jupp & Lawrence, 2010). In rats, N-acetylcysteine activates the cystine/glutamate-transporter, thereby increasing extrasynaptic glutamate levels and thus activating the mGluR2/3s which are downregulated after chronic cocaine-administration (Baker et al., 2003). Administration of N-acetylcysteine restores the GLT-1 levels, and this mechanism might be the most important factor determining the behavioural effects of the pharmaceutical (Reissner et al., 2015). By increasing the activity of the cystine/glutamate-transporter and restoring GLT-1 levels, N-acetylcysteine has been found to attenuate cue- (Reissner et al., 2015) and cocaine-induced reinstatement of cocaine seeking in rats (Baker et al., 2003). Interestingly, N-acetylcysteine also seems to influence the increased drug motivation seen in the withdrawal stage of addiction, as it has been found to decrease escalation of drug intake in rats with long access to cocaine (Madayag et al., 2007), although it does not seem to influence the maintenance of this behaviour nor the motivation of drug-seeking under a progressive-ratio-paradigm. Nevertheless, administration of N-acetylcysteine reduces cocaine-seeking in rats in the face of punishment (Ducret et al., 2016). In humans, administration of N-acetylcysteine to individuals addicted to cocaine significantly decreased cocaine craving and increased time to relapse (LaRowe et al., 2013). Furthermore, a review from 2017 based on randomized clinical trials found that N-acetylcysteine effectively reduced cravings of all the investigated drugs, including methamphetamine and cocaine (Duailibi et al., 2017). Of note, the ability of N-acetylcysteine to cross the blood-brain barrier has been questioned and seems to depend upon dose and administration (Bavarsad Shahripour et al., 2014), thereby questioning the mechanism underlying the above mentioned results. Nevertheless, the preliminary results incites investigations into pharmacokinetically superior N-acetylcysteine derivatives, such as N-acetylcysteine amide (Sunitha et al., 2013).

#### Ceftriaxone

Ceftriaxone is an approved beta-lactam-antibiotic used to treat certain kinds of bacterial infections. Like other beta-lactams it increases transcription of GLT-1 through an interaction with nuclear factor-kB (NF-kB) (Lee et al., 2008). Ceftriaxone has been shown to attenuate both cue- and drug-induced reinstatement in rats (Knackstedt et al., 2010). In addition, ceftriaxone has been shown to attenuate context-primed cocaine relapse in rats including increased levels of GluR1 levels and unchanged GluR2 levels in the nucleus accumbens without attenuating the relapse-dependent glutamate efflux of the nucleus accumbens (LaCrosse et al., 2016). Interestingly, when the reinstatement is cue-induced instead of context-induced, ceftriaxone attenuates the cocaine-seeking as well as the nucleus accumbens core glutamate efflux (Bechard et al., 2021). There are currently no ongoing clinal trials or earlier studies examining effects of ceftriaxone on addiction in humans. This could be because the antibiotic must be administrated intravenously, complicating the experimental setup.

Interestingly, it has been discovered that manipulation of the glutamate-homeostasis using either N-acetylcysteine or ceftriaxone induces enduring changes in drug-seeking behaviour in rats, as both drug-induced and cue-induced reinstatement are attenuated weeks after the therapeutics were given (Reichel et al., 2011; Sondheimer & Knackstedt, 2011), thus further highlighting the promise of these interventions.

### Summary of pharmacologic approaches targeting the glutamate system

The rationale for targeting molecular players in the glutamatergic transmission is to restore the complex maladaptive plasticity induced in the nucleus accumbens after repeated psychostimulant addiction as well as following drug abstinence. As such, the glutamatergic transmission is a critical target particular in late stages of addiction driven by habit and compulsive behaviour. While direct inhibition of AMPARs is highly challenging and associated with severe side-effects, modulation of the dynamic AMPAR trafficking by peptides that block intracellular scaffold proteins might be a more feasible therapeutic alternative, yet such approaches remain to be tested in clinical trials. Interference with metabotropic glutamate receptors in principle provides a more discrete mechanism for modulation and even reversal of the maladaptive plasticity induced by psychostimulants and may even be directed at different stages of addiction. Both antagonists and the positive allosteric inhibitors remain to be tested in clinical trials. Finally, reduced glutamate uptake is a central mechanism in maladaptive plasticity and several approved drugs can modulate and restore this deficit, with promising clinical trials for both ceftriaxone and N-acetylcysteine. For an overview of clinically tested drugs affecting the glutamate system mentioned in this review, see table 2.

**TABLE 2:** Human studies with pharmacological targeting of the glutamate system to treat psychostimulant addiction.

	Drug	References	Participants	Drug concentrations	Results
AMPA receptor	Topiramate	Kampman et al. (2004)	Placebo-controlled pilot study with 40 participants dependent on cocaine	Topiramate titrated over 8 weeks to 200 mg/day for 5 weeks	Topiramate-treated subjects were significantly more likely to be abstinent from cocaine compared to the placebo group
Indirect modulators of metabotropic glutamate receptor expression	N-acetylcysteine	LaRowe et al. (2013)	Placebo-controlled study with 111 cocaine-dependent volunteers.	Placebo, 1,200 mg or 2,400 mg N-acetylcysteine/day for 8 weeks	Only the subgroup of participants not using cocaine at start of trial had a positive effect of N-acetylcysteine with less craving and longer time to relapse

## Targeting withdrawal symptoms

An alternative approach to treat psychostimulant addiction is to target some of the withdrawal symptoms like increased stress, dysphoria, anxiety, decreased reward-sensation, and the compulsive drug-seeking (Koob et al., 2009).

### Corticotropin releasing factor antagonists

Corticotropin releasing factor (CRF) antagonists have been proposed as a future therapeutic due to the involvement of the neuropeptide CRF in several aspects of addiction, and especially its importance in withdrawal leading to increased CRF release in the amygdala of rats during withdrawal following cocaine self-administration (Richter & Weiss, 1999; Ubaldi et al., 2016). For instance, the selective CRF1 antagonists MPZP and CP-154,526 reduce cocaine self-administration in rats (Goeders & Guerin, 2000; Specio et al., 2008) and the non-selective CRF-antagonist D-phe CRF(12-41) minimize anxiogenic-like responses to withdrawal from chronic cocaine during the defensive burying paradigm but not in the elevated plus maze (Basso et al., 1999). Anxiety-like behaviour in the latter test was found to be reduced by administration of the selective CRF2-receptor antagonist antisauvagine-30 during methamphetamine withdrawal in rats (Reinbold et al., 2014). Furthermore, administration of the selective CRF1 receptor antagonist CP 154,526 attenuates increased cocaine-seeking during early extinction (withdrawal) in rats (Cason et al., 2016). CP 154,526 also attenuates cocaine-induced hyperlocomotion as well as cocaine-induced conditioned place preference and drug-induced reinstatement of cocaine seeking (Lu et al., 2003; Przegalinski et al., 2005). The non-selective CRF receptor antagonist α-helical CRF(9-41) can attenuate stress-induced reinstatement of methamphetamine-seeking in rats (Nawata et al., 2012). To our knowledge, no clinical trials with CRF antagonists have been performed for treatment of psychostimulant dependence specifically.

### Cognitive enhancers

Decreased cognitive abilities and impaired executive function are important parts of the relapsing nature of psychostimulant addiction (Goldstein & Volkow, 2011). By restoring these abilities in a person with addiction, the incomprehensible action of unintentional relapse could be halted. Moreover, a decrease in cognitive function has been linked to decreased treatment retention, another good reason to target this specific side-effect of addiction (Sofuoglu et al., 2016). It is mainly the noradrenergic, and cholinergic neurotransmitter systems that are being investigated as potential targets for enhancing cognition in psychostimulant addiction (Sofuoglu, 2010).

#### Atomoxetine

Atomoxetine is a selective noradrenaline reuptake inhibitor, used to treat ADHD-phenotypes like impulsivity and attentional impairment in humans. In rats, atomoxetine has been shown to decrease several distinct forms of impulsivity (Robinson et al., 2008). Furthermore, atomoxetine decreases the number of trials needed to reverse a learned discrimination in both a four-position discrimination task in rats and a three-choice visual discrimination task in monkeys (Seu et al., 2009).

In humans, a double-blind placebo-controlled study of a go/no-go task with cocaine-, and food-related stimuli, found that atomoxetine decreases the attentional bias of cocaine-dependent individuals (Passamonti et al., 2017). Another study found, that atomoxetine only has modest effects on cognition in an immediate memory test and shows no effect in a rapid visual processing test as well as in a stop signal task in individuals in early remission of cocaine addiction (DeVito et al., 2017a). Further analysis of the data indicated, that the lack of effect might be explained by sex differences, with the beneficial effects of atomoxetine only seen in males (DeVito et al., 2017b). The aspect of sex differences for the effects of atomoxetine has been seen in rodents as well (Smethells et al., 2016; Swalve et al., 2016). However, further research is needed to evaluate long-term treatment effects on psychostimulant intake and relapse.

#### Cholinesterase inhibitors

Cholinesterase inhibitors that are blood brain barrier permeable are currently used in humans to treat Alzheimer’s disease and are now being investigated in individuals addicted to psychostimulants as possible enhancers of cognitive function (Sofuoglu et al., 2016). In humans, pilot studies with the two cholinesterase inhibitors, galantamine and rivastigmine, have shown promising results in relation to treatment of cocaine addiction. Galantamine is a reversible competitive cholinesterase inhibitor with additional allosteric modulating properties of α7 and α4β2 nicotinic receptors, which in mice have been shown to improve spatial memory in a surgical model of memory impairment (Sweeney et al., 1988). Galantine has been shown to decrease cocaine intake and increase performance of abstinent cocaine users in the rapid visual information processing test, whereas rivastigmine increases working memory (Sofuoglu & Carroll, 2011; Sofuoglu et al., 2011; Mahoney et al., 2014). However, the results of a follow up clinical trial revealed no effect of galantamine on neither cocaine urine levels or cognitive function on patients with cocaine use disorder (DeVito et al., 2019).

## Discussion

**FIGURE 1:**
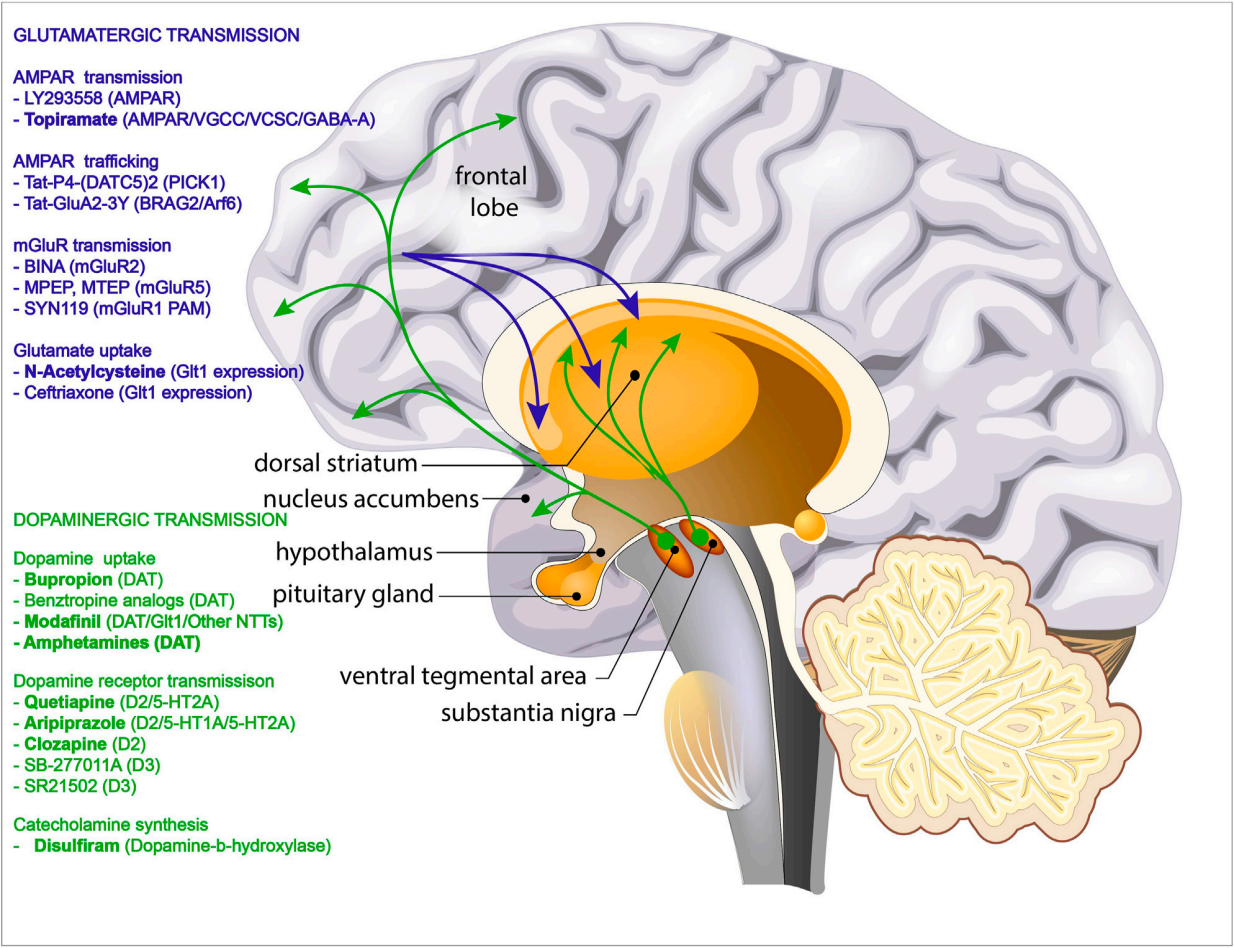
Overview of the pathways involved in psychostimulant addiction. Green arrows depict the mesocortical dopaminergic pathways projecting from the ventral tegmental area (VTA) to nucleus accumbens (NAc) and prefrontal cortex (PFC) and the mesolimbic dopaminergic pathways projecting from the substantia nigra to the dorsal striatum. Blue arrows depict the glutamatergic projections from the prefrontal cortex (PFC) into dorsal striatum and nucleus accumbens (NAc). Pharmacological agents that have been shown to reduce addictive behaviour by targeting the dopaminergic transmission are listed in green. Pharmacological agents that have been shown to reduce addictive behaviour by targeting the glutamatergic transmission are listed in green. Drugs that have been tested in human trials are shown in bold.

This review has presented results from investigations of a selection of pharmaceutical approaches for treatment of psychostimulant addiction, with some showing great promise. For an overview of the dopaminergic and glutamatergic pathways involved in psychostimulant addiction and the pharmacological agents shown to reduce addictive behaviour by targeting one of these pathways see Figure 1. Anecdotally one of the main issues when treating psychostimulant addiction is the craving seen during the first weeks of abstinence. Therefore, some of the most promising therapeutics might be the ones modulating the glutamate system and craving, with already approved pharmaceuticals such as n-acetylcysteine and ceftriaxone proving very intriguing. Since relapse can be initiated by context, any cue could be sufficient to induce relapse behaviour despite pharmacologically lowered reinforcing properties of a drug (Milton & Everitt, 2010). Nevertheless, since many of the therapeutics also regulate other aspects of addiction such as: compulsive drug-seeking (aripiprazole), extinction (D3-receptor antagonists), and reinstatement of drug-seeking (aripiprazole, bupropion, AHN-1055, D3-receptor antagonists, disulfiram), some of these pharmaceuticals might still hold a potential as future therapeutics. Each of the above-mentioned targets are problematic because of their non-selective molecular-directed actions, which give rise to certain drug-specific side effects, all complicating the transition from promising future therapeutic to approved clinical treatment.

The gap between the very promising results in well-founded animal models of addiction to low effect sizes in clinical trials is an important reason for the lack of approved treatment against psychostimulant addiction [for review see (Czoty et al., 2016)], along with issues regarding treatment retention and compliance (Morley et al., 2017), and the immense importance of social context in addiction (Heilig et al., 2016). When looking through pre-clinical data to find promising therapeutics for human trials of addiction, it is important to note how and why use of a drug is reduced in the specific animal models. If drug-use decreases because of side effects such as sedation, emesis, or aversion, efficacy will obviously diminish in humans because of lower compliance. A way to strengthen conclusions based on drug side effects, is to look for animal studies that include data on response to a non-drug reinforcer (i.e., food) in order to rule out these side effects (Czoty et al., 2016). Another way to approach the issue, is to reverse-translate successful clinical research in related fields to optimize pre-clinical research of treating psychostimulant use disorders (Venniro et al., 2020). This could lead to development of more and maybe even better pre-clinical models, but of course using such strategies involve the risk that new drugs will only make it to clinical trials, when their mechanism of action resemble the already existing ones. As mentioned in the introduction, some preclinical researchers have tried to better resemble the societal complexity of the human population with psychostimulant use disorders by adding environmental differences in the experimental setup, thereby influencing the vulnerability of increased drug intake of specific test subjects (Fouyssac et al., 2021).

It is also important to keep in mind that differences in treatment success between pre-clinical and clinical studies may be due in part to increased variability in behavioural phenotypes observed in human patients. Most clinical trials and subsequent meta-analysis lack two things; Firstly, they do not take phenotypes of the patients such as psychiatric comorbidity, frequency of drug use, genetic subgroups, and co-abuse of other drugs into account. For example, post-hoc analysis of a large clinical trial with Modafinil revealed significant effect of modafinil treatment only if the subgroup of comorbid alcohol users was removed from the analysis (Anderson et al., 2009). Secondly, they ignore factors related to differences in treatment strategies such as drug dosage, titration speed and formulation onset (Brandt et al., 2021). As different stages can dominate the symptoms in patients with psychostimulant disorders, discovering these differences and initial vulnerabilities toward addictive behaviour could present a possibility of more personalized treatment regimens and thereby increase efficacy of future therapeutics. For instance, different genotypes of the dopamine beta-hydroxylase might influence the effectiveness of disulfiram (Kosten et al., 2013) and polymorphism in CRF-receptor-genes have been associated with increased stress-reactivity and vulnerability to addictive behaviour (Ouzir & Errami, 2016), which could mean that treatment with CRF-antagonists could provide additional efficacy.

In general, proper stratification before initiation of clinical trials for substance use disorders is currently insufficient. Gender differences in responding to psychostimulants or psychostimulant-associated cues is well-known, and the same counts for differences between adolescents vs. adults (Kosten et al., 1996; Lynch et al., 2008; Wong et al., 2013; Runegaard et al., 2017; Caffino et al., 2021), yet many clinical trials base their analysis on mixed genders and wide age groups (see Tables 1, 2). The sex differences in treatment efficacy seen for both disulfiram, atomoxetine and bupropion highlight this need for stratification, since some of the negative treatment outcomes of clinical trials might stem from the combination of low patient numbers combined with mixed genders and random age groups being combined (Nich et al., 2004; Pani et al., 2010; Heinzerling et al., 2013; Heinzerling et al., 2014; DeVito et al., 2017b).

Another great pitfall of the clinical trials is patient compliance as well as the variation in outcome measures chosen. Modafinil, is an example where adequate adherence to treatment has been shown to be a necessity for the effectiveness of modafinil to treat psychostimulant disorder, confirming the need to validate medication compliance in clinical trials (Anderson et al., 2012). In relation to outcome measures, urine samples could help quantitatively determine levels of stimulant use during the clinical trial but is not always included. In general, a combination of self-report and biological indicators seem to be the proper primary outcome (Donovan et al., 2012).

Thus, the immense amount of research conducted in the field of neuroscience and addiction could someday provide effective and tolerable treatment for psychostimulant addiction and increase the translation from bench to bedside. Nevertheless, as addiction continues to be stigmatized, connected to shame, and include important psychological and social aspects, expensive new treatment might have long prospects. Therefore, accessible, and affordable therapeutics are also warranted. Finally, given that preclinical studies clearly demonstrate effects of targeting different pathways, an attractive clinical path would be to combine such drugs to meet the requirement of individual patients.
